# A case of pathological complete response with liposomal irinotecan + 5-FU/LV for unresectable locally advanced pancreatic cancer

**DOI:** 10.1186/s40792-022-01549-9

**Published:** 2022-10-07

**Authors:** Koji Kikuchi, Akira Umemura, Hiroyuki Nitta, Hirokatsu Katagiri, Masao Nishiya, Noriyuki Uesugi, Tamotsu Sugai, Keisuke Imanari, Akira Sasaki

**Affiliations:** 1 Department of Surgery, Iwate Medical University, 2-1-1 Idaidori, Yahaba, Iwate 028-3695 Japan; 2 Department of Molecular Diagnostic Pathology, Iwate Medical University, 2-1-1 Idaidori, Yahaba, Iwate 028-3695 Japan; 3 Department of Internal Medicine, Division of Gastroenterology, Iwate Medical University, 2-1-1 Idaidori, Yahaba, Iwate 028-3695 Japan

**Keywords:** Liposomal irinotecan, Pancreatic ductal adenocarcinoma, Unresectable pancreatic cancer, Pathological complete response

## Abstract

**Background:**

Pancreatic cancer has one of the worst prognoses of any all cancers. 5-FU/leucovorin + irinotecan + oxaliplatin (FOLFIRINOX), gemcitabine (GEM) plus nab-paclitaxel regimens have been recognized as global-standard, first-line treatments for patients with advanced pancreatic cancer. The liposomal irinotecan (nal-IRI) + 5-FU/LV regimen is now included in treatment guidelines as a recommended and approved option for use in patients with metastatic pancreatic cancer that has progressed after GEM-based therapy and who have a suitable performance status and comorbidity profile. There is no report that nal-IRI + 5-FU/LV regimen was significantly effective, and we will report it because we experienced this time.

**Case presentation:**

A 69-year-old man presented with epigastric pain, and a contrast computed tomography (CT) revealed an enhanced mass lesion measuring 33 × 27 mm on the pancreatic body with encasement of the common hepatic artery (CHA) and the splenic vein. An endoscopic ultrasound-guided fine needle aspiration was performed and demonstrated cytology consistent with adenocarcinoma. Therefore, we diagnosed the patient with unresectable locally advanced pancreatic cancer. The patient received the GEM and S-1 regimen; however, the adverse event was relatively severe. Then, 11 cycles of nal-IRI + 5-FU/LV regimen were administered. A CT scan revealed that the tumor had shrunk to 18 × 7 mm in diameter with encasement of the CHA. The encasement of the splenic vein had disappeared, without any distant metastases. From this post-chemotherapy evaluation and intraoperative frozen section of around the celiac artery, gastroduodenal artery and pancreas stump confirmed absence of tumor cells, we performed distal pancreatectomy with celiac axis resection. A histological examination of the surgical specimen revealed no evidence of residual adenocarcinoma, consistent with a pathological complete response to treatment.

**Conclusions:**

We present the first case of a pathological complete response with nal-IRI + 5-FU/LV for unresectable, locally advanced pancreatic cancer. In the future, nal-IRI may become a key drug for pancreatic cancer treatment.

## Background

Pancreatic cancer has one of the worst prognoses of all cancers. Only 15–20% of cases are resectable, 30–40% of cases involve locally advanced (LA) cancer, and 50–60% of cases involve distant metastatic cancer, which is unresectable (UR) [1]. Systemic chemotherapy is employed as the standard of care for UR pancreatic cancer in both LA and metastatic diseases. The 2019 Clinical Practice Guidelines for Pancreatic Cancer suggest that conversion surgery after multidisciplinary treatment could be a treatment option for UR-LA pancreatic cancer because favorable overall survival (OS) and/or progression-free survival (PFS) can be expected [2, 3]. In a retrospective multicenter study involving 97 patients with UR-LA pancreatic cancer in Japan, conversion surgery was more beneficial for patients with more than 8 months of preoperative therapy than those with less than 8 months of that therapy [4].

Although systemic chemotherapy with gemcitabine has been the standard of care for advanced pancreatic cancer since 1997, the efficacy of gemcitabine (GEM) has not been satisfactory [5]. FOLFIRINOX (5-FU/leucovorin + irinotecan + oxaliplatin) and the GEM plus nab-paclitaxel (GNP) regimens provide significant survival benefits over gemcitabine monotherapy and have recently been recognized as global-standard, first-line treatments for patients with advanced pancreatic cancer [6–9]. In addition, S-1 monotherapy has been shown to achieve a significantly better response rate and non-inferior overall survival against GEM (when used alone) for advanced pancreatic cancer patients in the randomized Phase III GEM and S-1 trial [10]. For its favorable safety profile, S-1 either alone or combined with GEM is an acceptable regimen for less fit advanced pancreatic cancer patients in Japan [9, 11].

In the global NAPOLI-1 Phase III trial, Liposomal Irinotecan (nal-IRI) + 5-FU/LV significantly increased median OS versus 5-FU/LV (median OS: 6.1 months vs. 4.2 months; unstratified hazard ratio [HR] = 0.67; *p* = 0.012) in patients with metastatic pancreatic cancer that progressed after GEM-based therapy. The median investigator-assessed PFS was also improved in these patients (3.1 months vs. 1.5 months; HR = 0.56; *p* = 0.0001) [12]. The nal-IRI + 5-FU/LV regimen is now included in treatment guidelines as a recommended and approved option for use in patients with metastatic pancreatic cancer that progressed after GEM-based therapy and who have a suitable performance status and comorbidity profile [13–15].

In this report, we present a case of pathological complete response (CR) with nal-IRI + 5-FU/LV for UR-LA pancreatic cancer. This is the first report in which pathological CR has been identified in patients who were treated with nal-IRI + 5-FU/LV.

## Case presentation

**Fig. 1:**
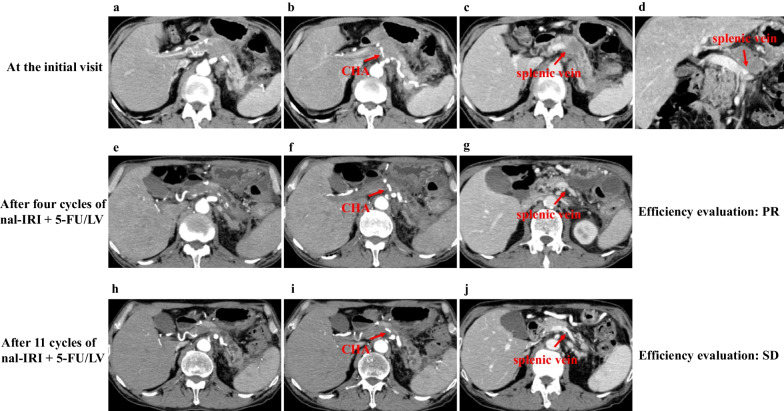
Consecutive CT findings from at baseline to preoperative point. **a**–**d** An enhanced mass lesion measuring 33 × 27 mm on the pancreatic body with encasement of the CHA and narrowing of the splenic vein at the initial visit. **e**–**g** The tumor shrank to 25 × 10 mm in diameter with the encasement of the CHA and narrowing of the splenic vein after four cycles of liposomal irinotecan + 5-FU/LV chemotherapy regimen administered. **h**–**j** The tumor shrank to 18 × 7 mm in diameter with the encasement of the CHA. The narrowing of the splenic vein disappeared after 11 cycles of liposomal irinotecan + 5-FU/LV were administered

**Fig. 2:**
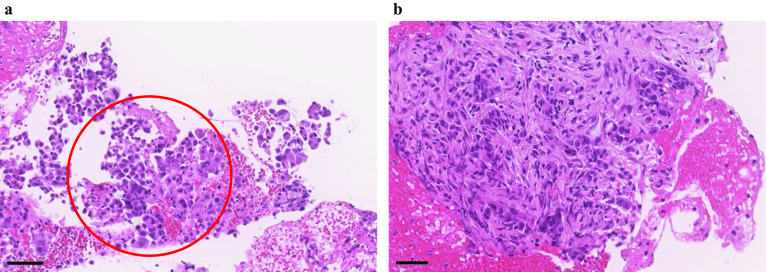
Histopathological findings of EUS-FNA. **a** Atypical cells showing nuclear enlargement proliferate without forming ducts (HE stain, scale bar; 60 μm). It is a poorly differentiated type. **b** The interstitial infiltration of atypical cells is observed (HE stain, scale bar; 40 μm)

A 69-year-old man presented with epigastric pain, and his past medical history included gastric ulcers and alcoholic pancreatitis. Contrast computed tomography (CT) revealed an enhanced mass lesion measuring 33 × 27 mm on the pancreatic body with encasement of the common hepatic artery (CHA) and narrowing of the splenic vein; there was no distant metastasis (Fig. 1a–d). A malignant pancreatic tumor was suspected, and an endoscopic ultrasound-guided fine needle aspiration (EUS-FNA) of the lesion was performed that demonstrated cytology consistent with pancreatic ductal adenocarcinoma (PDAC) (Fig. 2). Therefore, we diagnosed UR-LA pancreatic cancer.

Based on these examinations, we planned to perform doublet chemotherapy of GEM and S-1 because the supply of nab-paclitaxel has dwindled since August 2021 and this patient was found to be double heterozygous for *uridine-diphosphate glucuronosyltransferase* 1A1 (UGT1A1) *28 and UGT1A1*6. FOLFIRINOX has not dose adjustment criteria for patients with double heterozygous for UGT1A1*28 and UGT1A1*6. Besides, in the Phase II study of FOLFIRINOX for chemotherapy-naïve Japanese patients with metastatic pancreatic cancer, patients were excluded if they had UGT genetic polymorphisms heterozygous UGT1A1*6 and UGT1A1*28 [16]. For these reasons, the choice of FOLFIRINOX was not easy for patients with double heterozygous for UGT1A1*28 and UGT1A1*6. The patient received GEM (1000 mg/m^2^) on days 1 and 8, and oral S-1 [(100 mg/day (BSA < 1.5m^2^)] on days 1–14. This treatment was repeated at 21-day intervals. After the first cycles of GEM and S-1, this regimen was discontinued due to exanthematous drug eruption as a side effect of GEM. Because this patient was found to be double heterozygous for UGT1A1*28 and UGT1A1*6, and S-1 was used in the first-line treatment, the second-line treatment entailed the administration of nal-IRI + 5-FU/LV (80 mg nal-IRI, 4000 mg fluorouracil, and 340 mg leucovorin) in 11 cycles. The starting dose for nal-IRI was reduced to 70% according to dose adjustment criteria for patients with double heterozygous for UGT1A1*28 and UGT1A1*6. The treatment was repeated at 14-day intervals.

**Fig. 3:**
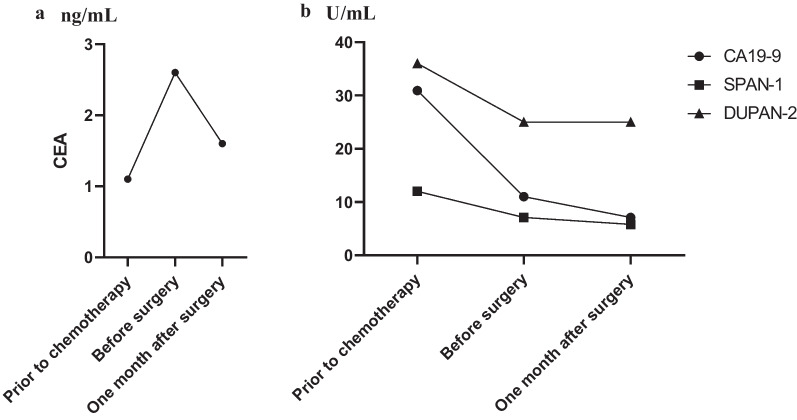
Changes in tumor markers. **a** Changes in CEA. **b** Changes in CA19-9, SPAN-1 and DUPAN-2. CEA, carcinoembryonic antigen; CA19-9, carbohydrate antigen 19-9; SPAN-1, s-pancreas antigen-1; DUPAN-2, duke pancreatic monoclonal antigen type 2

**Fig. 4:**
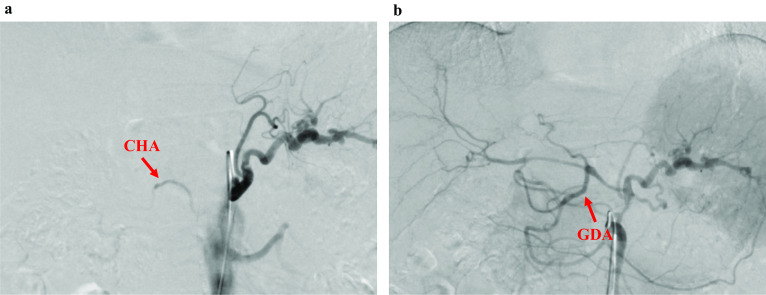
Preoperative angiography. **a** When the celiac artery was imaged, only the proximal part of the CHA was visible. **b** When the superior mesenteric artery was imaged, the hepatic blood flow occurred predominantly from the superior mesenteric artery through the GDA

**Fig. 5:**
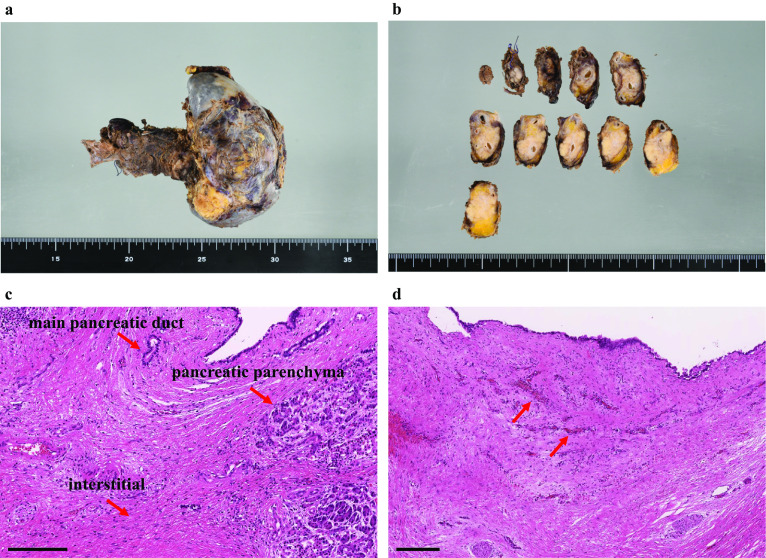
Histopathological findings of resected specimens. **a**, **b** Macroscopically, no mass lesions were found in the pancreas. The atrophy of the pancreatic parenchyma and an increase in fibrillar connective tissue were observed. **c** The fibrosis of the main pancreatic duct, pancreatic parenchyma, and the interstitial was confirmed (HE stain, scale bar; 200 μm). **d** There was an increase in small blood vessels, and it is possible that cancer was once here (HE stain, scale bar; 500 μm)

After four cycles of nal-IRI + 5-FU/LV were administered, CT revealed that the tumor had shrunk to 25 × 10 mm in diameter, with encasement of the CHA and narrowing of the splenic vein (Fig. 1e–g). This was considered a partial response to the nal-IRI + 5-FU/LV chemotherapy regimen according to the Response Evaluation Criteria in Solid Tumors (RECIST) guidelines, Ver. 1.1 [17]. After 11 cycles of nal-IRI + 5-FU/LV chemotherapy regimen were administered, CT revealed that the tumor had shrunk to 18 × 7 mm in diameter with encasement of the CHA. The narrowing of the splenic vein had disappeared, and there was no distant metastasis (Fig. 1h–j). This was considered a stable disease to nal-IRI + 5-FU/LV chemotherapy regimen according to the RECIST guidelines [17]. There were not any severe adverse events except for grade 1 anorexia. The tumor markers including the carcinoembryonic antigen, carbohydrate antigen 19-9, s-pancreas antigen-1 and duke pancreatic monoclonal antigen type 2, were normal from the start of treatment (Fig. 3a, b). We performed angiography for the purpose of preoperative embolization of the CHA for arterial redistribution. When the celiac artery was imaged, however, only the proximal part of the CHA was imaged (Fig. 4a). The hepatic blood flowed predominantly from the superior mesenteric artery through the gastroduodenal artery (GDA) (Fig. 4b); thus, embolization of the CHA was not performed. Intraoperative histopathological findings of frozen section confirmed tumor-free margins of the celiac artery, GDA and pancreas stump. Based on these findings, a distal pancreatectomy with celiac axis resection, including the left gastric artery, was performed 17 days after the final chemotherapy. There was no involvement of the superior mesenteric vein and portal vein. Thus, we did not perform any vascular reconstruction. The operating time and blood loss were 488 min and 275 mL, respectively. A histological examination of the surgical specimen revealed no evidence of residual adenocarcinoma including co-resected lymph nodes. Macroscopically, no mass lesions were found in the pancreas, and atrophy of the pancreatic parenchyma and an increase in fibrillar connective tissue were observed (Fig. 5a, b). The fibrosis of the main pancreatic duct, pancreatic parenchyma, and the interstitial was confirmed (Fig. 5c) and there was an increase in small blood vessels; thus, it is possible that cancer was once present (Fig. 5d). This finding is consistent with a pathological CR to treatment (Evans Regression Score grade III) [18]. The postoperative course was unremarkable, and the patient was discharged on postoperative day 12. The patient received adjuvant chemotherapy with S-1 and remains disease-free 5 months following surgery.

## Discussion

To the best of our knowledge, this is the first case in which pathological CR has been identified in a patient who was treated with nal-IRI + 5-FU/LV. CR is rarely observed in patients with PDAC who receive neoadjuvant therapy and pancreatectomy. Before the emergence of FOLFIRINOX and the gemcitabine plus nab-paclitaxel regimens in the early 2010s, a systematic review including 111 retrospective and prospective studies analyzing neoadjuvant radiochemotherapy, radiotherapy, or chemotherapy of pancreatic cancer patients (n = 4394) reported that average radiological and pathological CR probabilities were 3.9% [1]. According to a recent study, the final pathological review revealed pathological CR in 18.5% of initially UR pancreatic cancer patients who underwent conversion surgery following induction therapy [19]. However, in each systematic review of neoadjuvant FOLFIRINOX and GNP for pancreatic cancer, it was reported that pathological CR had been seen in 4.4% of patients who received neoadjuvant FOLFIRINOX alone or combined with radiotherapy, and no patient showed radiological CR after therapy with GNP [20, 21]. Even in recent reports, there are variations in the CR rate.

The prediction of resection possibility by CT after preoperative treatment is unreliable. It was reported that preoperative CT revealed that 70% of patients retained UR-LA/BR pancreatic cancer, but R0 resection was possible in 92% of patients [13]. Instead, local resectability can only be assessed by surgical exploration, with frozen section biopsies. If the frozen biopsy reveals a persistent true invasion of a major artery, surgical resection can be abandoned, or the decision for an arterial resection must be made [22]. In our case, however, a preoperative CT revealed that the tumor had shrunk to 18 × 7 mm in diameter with encasement of the CHA, so the findings of UR-LA pancreatic cancer remained, and intraoperative frozen section pathology findings confirmed tumor-free margins. This suggests that it is dangerous to judge whether pancreatic cancer is resectable only by CT, and complex judgment is required.

In the global NAPOLI-1 Phase III trial, the grade 3 or 4 adverse events that occurred most frequently in the patients assigned nal-IRI + 5-FU/LV were neutropenia (27%), diarrhea (13%), vomiting (11%), and fatigue (14%) [12]. In the present case, however, there were not any severe adverse events except for grade 1 anorexia. Nal-IRI + 5-FU/LV is currently positioned as a second-line treatment after GEM-based therapy or FOLFIRINOX. Therefore, there are few reports of conversion surgery after the nal-IRI + 5-FU/LV regimen. A phase 1/2 study in previously untreated LA/metastatic pancreatic cancer showed promising anti-tumor activity with nal-IRI 50 mg/m^2^ free base + 5-FU 2400 mg/m^2^ + LV 400 mg/m^2^ + oxaliplatin 60 mg/m^2^ as a NALIRIFOX regimen on days 1 and 15 of a 28-day cycle [23]. Hence, clinical trials in which randomized, Phase III study of first-line NALIRIFOX versus gemcitabine plus nab-paclitaxel in, patients with metastatic pancreatic cancer are in progress. Depending on the results of this clinical trials, nal-IRI may be used as the first line of pancreatic cancer treatment.

## Conclusion

In this paper, we present the first case of pathological CR with nal-IRI + 5-FU/LV for UR-LA pancreatic cancer. In the future, nal-IRI may become a key drug for pancreatic cancer treatment.

## Data Availability

Not applicable.

## Abbreviations

LA: Locally advanced
UR: Unresectable
OS: Overall survival
PFS: Progression-free survival
GEM: Gemcitabine
GNP: GEM plus nab-paclitaxel
HR: Hazard ratio
CR: Complete response
CT: Computed tomography
CHA: Common hepatic artery
EUS-FNA: Endoscopic ultrasound-guided fine needle aspiration
PDAC: Pancreatic ductal adenocarcinoma
UGT1A1: *Uridine-diphosphate glucuronosyltransferase* 1A1
RECIST: Response Evaluation Criteria in Solid Tumors
GDA: Gastroduodenal artery
